# A Comparison of Osteoblast and Osteoclast In Vitro Co-Culture Models and Their Translation for Preclinical Drug Testing Applications

**DOI:** 10.3390/ijms21030912

**Published:** 2020-01-30

**Authors:** Alexander Sieberath, Elena Della Bella, Ana Marina Ferreira, Piergiorgio Gentile, David Eglin, Kenny Dalgarno

**Affiliations:** 1 School of Engineering, Newcastle University, Newcastle-Upon-Tyne NE1 7RU, UK; a.sieberath2@newcastle.ac.uk (A.S.); ana.ferreira-duarte@ncl.ac.uk (A.M.F.); piergiorgio.gentile@ncl.ac.uk (P.G.); 2 AO Research Institute Davos, Clavadelerstrasse 8, 7270 Davos, Switzerland; Elena.DellaBella@aofoundation.org (E.D.B.); david.eglin@aofoundation.org (D.E.)

**Keywords:** co-culture, osteoblast, osteoclast, in vitro model, drug testing, osteoporosis

## Abstract

As the population of western societies on average ages, the number of people affected by bone remodeling-associated diseases such as osteoporosis continues to increase. The development of new therapeutics is hampered by the high failure rates of drug candidates during clinical testing, which is in part due to the poor predictive character of animal models during preclinical drug testing. Co-culture models of osteoblasts and osteoclasts offer an alternative to animal testing and are considered to have the potential to improve drug development processes in the future. However, a robust, scalable, and reproducible 3D model combining osteoblasts and osteoclasts for preclinical drug testing purposes has not been developed to date. Here we review various types of osteoblast–osteoclast co-culture models and outline the remaining obstacles that must be overcome for their successful translation.

## 1. Introduction

Bone undergoes lifelong modelling and remodelling processes. During bone modelling, the bone shape (e.g., trabecular orientation, thickening/thinning of the compact bone layer) is optimised according to the experienced loading forces on the bone, while bone remodelling resembles a homeostatic process in which bone strength and mineral content is maintained and balanced [1]. This is achieved by the resorption of bone matrix by osteoclasts and the deposition of fresh bone matrix called osteoid by osteoblasts. Thus, osteoclasts and osteoblasts are key players during bone turnover, and their actions are tightly intertwined during this process.

**Figure 1:**
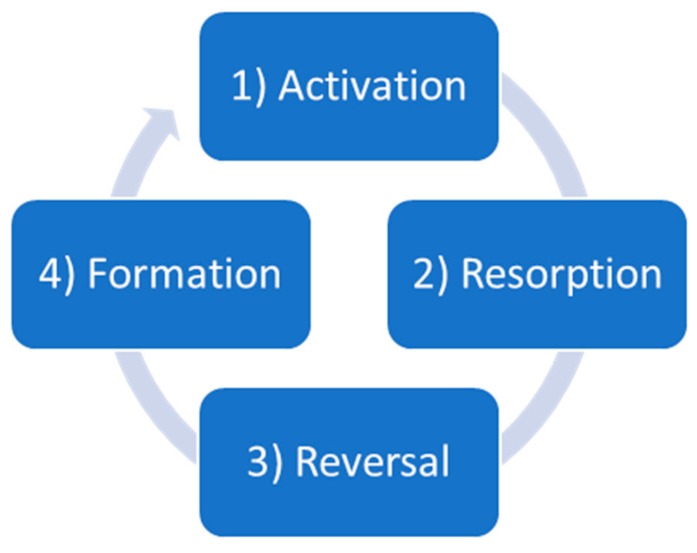
Sequential remodelling in cancellous bone.

Under homeostatic circumstances, the amount of resorbed bone matrix by osteoclasts equals the amount of newly synthesised bone matrix by osteoblasts [2], and this process is performed sequentially in 4 steps [1,3] (Figure 1):

- Osteoclast precursors are recruited from the bloodstream and form multinucleated osteoclasts (activation) [1];
- Mature osteoclasts resorb bone matrix through the acidification of the extracellular environment with the help of a proton pump [4];
- Osteoclasts undergo apoptosis, and pre-osteoblasts are recruited to the resorption site (reversal) [1];
- Deposition (formation) of fresh unmineralised matrix called osteoid by osteoblasts and subsequent mineralisation [5].

**Figure 2:**
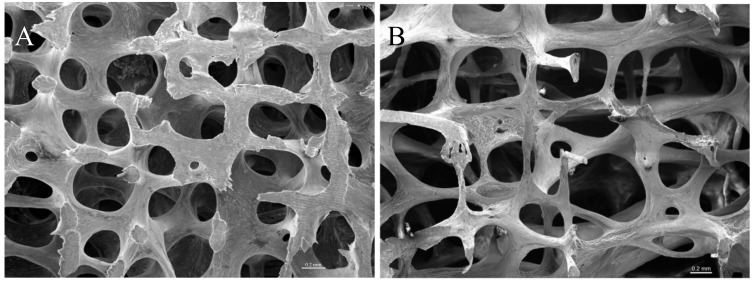
Scanning electron micrographs of healthy (**A**) and osteoporotic (**B**) trabecular bone. The osteoporotic bone shows increased porosity in comparison to healthy bone. The increased pore size weakens the mechanical properties and consequently leads to fracture of the bone (reproduced with permission of Prof. Timothy Arnett, University College London; original image available at boneresearchsociety.org).

Approximately 10% of total bone mass is remodelled every year in a healthy adult [5]. While bone synthesis resembles a more complex process and thus takes up to 6 months, breakdown of bone tissue is rather fast and is completed in about 2 to 4 weeks [1]. Consequently, any changes in the synthesis- and/or resorption rates critically affect bone homeostasis and are a major cause of bone diseases such as osteoporosis. Osteoporosis becomes more common in older people and is characterised by a reduced bone mass, impaired bone quality, and a propensity to fracture as a result of an imbalance between bone formation and resorption [2]. The majority of the observed bone loss occurs in the trabecular bone by progressive thinning of the trabeculae, which leads to increased porosity and thus declining mechanical strength of the affected bone (Figure 2).

Thus, fractures associated with osteoporosis occur frequently in anatomical locations like the femoral neck, the vertebrae, or the radius, which are dominated by trabecular bone [6]. Fractures at the hip are especially vicious as 30% of elderly patients (older than 65 years) die within the following years after a fracture due to their loss of mobility [7]. In addition to the individual burden, osteoporosis also represents a significant economic issue in health care systems all over the world. For example, in the UK, the expected annual cost for osteoporosis associated health care will rise by 30% until 2030 to £5.89 billion [8]. Most of the existing osteoporosis therapies include the use of antiresorptive drugs such as bisphosphonates, estrogen and estrogen-receptor modulators. The majority of these agents reduce the rate of bone loss through the inhibition of osteoclast activity or differentiation, but they do not promote bone synthesis [9]. The only approved exception so far is the parathyroid hormone substitute, Teriparatide, which supports for the formation of new bone by the activation of osteoblasts, but its application is limited to two years due to increased risk of osteosarcoma development [10]. Moreover, the prescription rates of bisphosphonate-based drugs such as alendronic acid have fallen significantly due to the increased risk of atypical femoral fractures and osteonecrosis of the jaw [2].

So far, preclinical drug testing relies heavily on animal models of osteoporosis [11,12,13], which enable researchers to study the effects of specific substances on bone metabolism on a systemic level. The three main types of osteoporosis in humans (postmenopausal osteoporosis, disuse osteoporosis, and glucocorticoid-induced osteoporosis) can be modelled to some extent in animals, allowing researchers to investigate the disease subtype of interest [14]. Table 1 summarizes the main osteoporosis animal models currently validated. The most prominent model of postmenopausal osteoporosis is induced by ovariectomy while the model for disuse osteoporosis is generated by the immobilisation of the animal which can, for instance, be achieved by sciatic neurectomy or tail suspension. Glucocorticoid-induced osteoporosis, found in humans taking drugs such as dexamethasone or prednisone, can also be generated by treating animals with glucocorticoids (e.g., by oral dosage forms or infusions).

The meaningfulness of osteoporosis studies using animal models is strongly related to how closely the animal model is to replicating the pathological human situation. However, these in vivo models suffer from species-specific differences in terms of bone composition, density and quality, which reduces their significance for translational research [15]. It has, for instance, been shown that rats, which are the most commonly used animal model in orthopaedic research [16], lack the well-developed haversian remodelling system [17] that is present in humans. Furthermore, it has to be considered that only human- and non-human primates are able to suffer naturally from osteoporosis, but next to the ethical concerns, the acquisition as well as the species-appropriate husbandry of primates is expensive [18]. It is only possible to induce an osteoporotic-like state by ovariectomy, immobilization or by drug treatment as in the other commonly used model organisms [19]. This forced induction of the osteoporotic state leads to notable differences between the observed human and animal pathophysiology [18] and thus does not recapitulate the clinical course of the disease. For example, ovariectomy in sheep causes an increase in porosity of compact bone but has no effect on the trabecular bone structures after six months [20]. On the contrary, postmenopausal osteoporosis is defined by a major loss of trabecular bone mass in humans. Compared to the clinical situation, the aforementioned model organisms also lack the range of specific biomarkers to assess bone metabolism accurately [17]. Even though reagents designed for human bone diagnostics can be adapted for their use in animals, this process is often accompanied by a loss of specificity and sensitivity of the respective compound [18]. Another factor to consider is that these biomarkers of bone turnover only measure changes on the whole skeleton level and do not allow us to track changes on the tissue level (meaning whether changes in bone remodelling occur in the compact or trabecular bone compartment) [18]. Moreover, the animal body itself poses an obstacle for clear imaging of the therapeutic agent within tissues or organs, limiting the options for applicable visual readouts [21]. In the European Union (EU), Directive 2010/63/EU regulates how animal research is performed, largely based on the 3R principle (replacement, reduction, refinement). This precept was developed by Russel and Burch [22] and states that the number of animals for experiments and their burden should be reduced to a minimum, and if possible, be replaced by in vitro models. However, health authorities (such as the Federal Drug Agency in the US and the European Medicines Agency) require pharmacological companies to test their osteoporosis drug candidates in ovariectomised rats and one larger animal model before proceeding to the clinical stage [12,13]. During animal testing, candidates are benchmarked concerning their effect on bone mineral density, bone turnover markers and mechanical testing [23].

All in all, the use of animal models for preclinical drug testing is connected with ethical concerns, comparable high cost and low throughput [24], and their results are not predictive for human outcomes. This is indicated by the fact that less than 10% of the drugs entering clinical testing, and thus have been successfully tested in animals, are, in the end, approved by the health authorities [25]. New treatments for osteoporosis that support bone synthesis will require improved disease models, and this paper will provide an overview of osteoblast–osteoclast co-culture models that have been proposed for use in osteoporotic drug development.

## 2. In Vitro Co-Culture Models of Osteoblasts and Osteoclasts

Due to the shortcomings of osteoporosis animal models, researchers have focused on the development of in vitro models using cell lines or primary cells to gain more relevant preclinical research results. In vitro models allow the use of human cells and focus on a restricted number of biological interactions and mechanisms as they usually represent a simplistic representation of the in vivo situation. In general, in vitro bone models of bone can be considered in three main categories:

1. Primary bone tissue culture—bone tissue explants are cultured in their physiological tissue architecture.
2. 2D bone cell cultures—cells, extracted from bone tissue or immortalised cell lines, are cultured in 2D monolayers on plastic cell culture dishes or other flat substrates.
3. 3D bone cell cultures—cells, extracted from bone tissue or immortalised cell lines, are cultured in a 3D environment by seeding them on scaffolds, matrices or as spheroids.

Many of these models within the three categories aim to recapitulate the remodelling process due to its fundamental role in bone biology and its associated diseases. These models are either set up using one (monocultures) or multiple bone-related cell types. Here, the use of multiple cell types is advantageous as direct and indirect cell interactions between two cell types (or more) are included, which consequently leads to a better representation of the in vivo than in monocultures. Due to their major role during bone formation and resorption, the co-culture of osteoblasts and osteoclasts is a promising approach for the development of new in vitro models, which could potentially deliver more predictive preclinical drug testing results than animal models.

Here, we review the different types of osteoblast/osteoclast co-culture models and discuss their advantages and disadvantages for preclinical drug screening.

### 2.1. Bone Tissue Cultures

Cultures of bone tissue explants are setup by first thinly slicing bone specimens. The bone slice is then cultured, with the thin slicing necessary to allow nutrient and gas diffusion during culture. Using this methodology, bone cells remain in their physiological organisation and thus this approach very closely replicates the in vivo situation. Table 2 summarizes how bone tissue cultures have been used in the literature.

#### 2.1.1. Culture Types

Several studies have focused primarily on bone culture method development to address specific research questions. Many of the studies involved static cultures, for example, with the use of murine embryonic metatarsals as a physiological model of endochondral ossification [41], chick femurs for organotypic cultures to study skeletal development [42], or different murine/bovine bone types to assess linear bone growth, bone and cartilage metabolism, bone response to mechanical stimulation, or bone resorption and formation [43]. Other static culture models were also developed to study inflammatory bone destruction [44] or the effect of molecules on gene expression levels [45].

More complex culture types have also been developed. Davidson et al. and Swarup and colleagues [39,40] both employed perfusion bioreactors for better preservation of bone viability when cultured ex vivo. In the first case, the cultures of rat femurs were maintained for 14 days, while in the second case, bovine or human femoral heads were cultured for only 12 hours, studying the effect of medium supplementation with heparin or mannitol to improve tissue perfusion. A 3D load-providing perfusion culture system (ZetOS, [48,49]) was developed and was able to maintain viability of bone tissue for up to 2–3 weeks of culture, and was suggested as a method for studying basic bone biology and mechanobiology, biomaterials interactions, or the effect of treatments that impact on bone remodelling processes. This culture method has the great benefit of including mechanical load, which is often overlooked but is an essential parameter to consider in the complex equation of bone physiology.

#### 2.1.2. Bone Formation and Remodelling in Bone Tissue Culture Models

Different models were used to assess bone formation and remodelling in ex vivo cultures, with metatarsal or calvarial bone cultures often used as models of endochondral or intramembranous bone formation, respectively. Models have been used to study signalling components or pathways or even circadian rhythms in bone/cartilage development [53,57,78,79,80,81].

Liu et al. [61] proposed murine calvarial bone culture models to dissociate bone resorption and formation to study the biological functions of egg protein phosvitin. The effect of phosvitin on bone resorption was studied under PTH treatment in an ascorbate-free environment, while bone formation was assessed in either the absence or presence of ascorbate. The results led to the conclusion that phosvitin mirrors the action of ascorbic acid in bone formation and potently inhibits bone resorption.

The effect of different treatments was also studied using bone tissue cultures. In 1999, different antisense DNA (targeting different subunits of the osteoclastic vacuolar H^+^-ATPase) were used as treatments to inhibit resorption in cultured bones [62], and decreased osteoclast formation by TRAP staining was observed. Bodine et al. [63] used mouse calvarial bones to assess the effect of secreted frizzled-related protein (sFRP-1) inhibitors (an antagonist of Wnt signalling). The authors assessed bone formation by histomorphometric analysis, determining total bone area and the number of osteoblasts. This method proved suitable for studying the effect of small molecules on bone formation. A similar approach was used for a viral-induced overexpression or knock-down of the Ror2 gene [64]. Nakajima et al. [65] used both metatarsal or calvarial bone cultures (7 days or 2 weeks of culture, respectively) in the presence or absence of a helioxanthin derivative. A pro-osteogenic effect of the compound on both models of bone formation was observed, as assessed by Von Kossa staining and by the number of osteoblast-like cells. In ex vivo cultures of craniomaxillofacial bone from Atlantic cod, the effect of retinoic acid on bone remodelling was assessed mainly by gene expression of osteoblastic or osteoclastic markers [66]. Also, the effect of culturing bones ex vivo in the presence PTH was assessed by qPCR analysis, with a particular focus on RANKL, sclerostin and osteoprotegerin regulation [67].

As for bone repair, one paper reported a mandible culture model [46], with slices of mandibles with fractures cultured up to 21 days in static culture with bones embedded in an agarose gel. Other models included chick femur organotypic cultures to assess the effect of growth factor delivery via hydrogels on bone repair [54,55] or to study skeletal development [42,82,83]. Chagin et al [68] developed long term cultures of fetal or postnatal rat bones to analyse the effect of dexamethasone withdrawal on bone growth. It is worth mentioning that the authors were able to maintain the bones in static cultures for three months using DMEM-F12 (supplemented with β-glycerol phosphate, ascorbic acid and bovine serum albumin), observing a continuous growth, even if this slowed towards the end of the culture period. The effect of organophosphate ester (used in flame retardants) on endochondral ossification was assessed in a murine limb bud culture system, with bones derived from transgenic mice expressing Col2a1-ECFP, Col10a1-mCherry, and Col1a1-YFP [56]. The bones cultured for six days in the presence of the compounds showed that reporter protein expression was impaired, as well as the expression of endochondral ossification gene expression markers.

Interestingly, only one paper dealt with bone infection and bone resorption [58]. The authors showed that *S. aureus* can stimulate bone resorption in ex vivo cultures of mouse parietal bones, as determined by the release of 45Ca and bone matrix degradation fragments in the culture medium. This effect was abolished by bisphosphonate treatment. Gene expression analysis showed an increase in osteoclast marker expression upon *S. aureus* infection.

Different works focused on the effect of load or other mechanical or biophysical stimuli [59,69,71,72,73,74,75,76,77]. Among others, David et al. [59] assessed in vivo bone formation in the ZetOS culture system. Osteocyte viability was assessed by LDH staining, ALP activity measured in medium and bone, while RUNX2 and osteocalcin protein and RNA expression were measured by ELISA and qPCR, respectively. The results showed that mechanical strain stimulates bone formation, and, in this system, osteoclasts can respond to drugs like retinoic acid or bisphosphonates. In another model, rat mandibular slices were cultured up to seven days with applied compressive forces to simulate conditions of orthodontically induced root resorption. The number of osteoclasts and odontoclasts were assessed by histology and dental sialoprotein expression was determined [47].

The maintenance of full tissues or organs in ex vivo culture might be a challenging task, but tissue cultures may provide a unique platform for the study of bone remodelling in the context of cell–cell interaction in their native environment. In addition, they represent a valuable tool for the application of 3Rs principles. There are a variety of established readout methods including viability, histology and histomorphometry, analysis of gene expression, and the determination of calcium release or enzyme activity.

However, the limited availability of human bone is one of the biggest disadvantages of this model type. Out of all the studies reviewed above, only five utilised bone specimens of human origin. Therefore, many rely on the use of animal-derived specimens, which suffer from known species-specific differences in bone and progenitor cell biology (e.g., BMP response of progenitor cells differs significantly in rat and human systems [70]). Further major drawbacks are reduced reproducibility due to donor variability and low throughput due to the complex manual model setup.

#### 2.2. 2D Co-Culture Models

2D co-culture models of bone-derived cells are usually set up using standard cell culture vessels and thus are rather simple and cost-effective. This model type can be direct contact or indirect, run in either static or dynamic mode.

#### 2.2.1. 2D Indirect Co-Culture Models

In indirect models, osteoblasts and osteoclasts are spatially divided using spacers such as transwell inserts [84,85,86,87,88,89,90], semi-permeable membranes [91,92] or by swapping conditioned medium [90,93,94,95,96] between the cell types. Indirect models allow the study of the paracrine effects of osteoblast and osteoclasts, such as the release of cytokines, extracellular vesicles or microRNAs [97].

Kubota et al used a conditioned medium of osteoclast-like RAW264.7 cells to evaluate their effect on the osteoblast differentiation of MC3T3-E1 cells. They found that the differentiated RAW264.7 supressed osteoblastogenesis, as suggested by reduced ALP activity or by the release of the platelet-derived growth factor BB [93]. However, the authors did not investigate if there was a feedback signal of osteoblasts treated with platelet-derived growth factor to modulate osteoclasts in return.

Bernhardt et al developed a more complex indirect transwell co-culture model by supplying osteoblast and osteoclast precursors with a bone-like microenvironment [84]. This was achieved by culturing primary hMSCs and hPBMCs on hydroxyapatite type I collagen composite membranes. Using this model, the authors investigated the paracrine effects of both cell types on each other during simultaneous osteoblast and osteoclast differentiation. They showed that during indirect co-culture, the osteoblastic ALP activity, as well as its gene expression levels, were enhanced in osteogenically induced hMSCs while osteoclastic differentiation was suppressed (measured by reduced tartrate-resistant acid phosphatase (TRAP) activity). Even though Bernhardt et al analysed the osteoclast differentiation on the bone-like substrate in terms of TRAP expression and activity, they did not assess the functionality of the cells (meaning matrix resorption). Also, they did not compare the performance of the cells on the composite substrate vs. co-cultures on conventional cell culture dishes to evaluate the effect of the provided microenvironment.

In contrast to static 2D co-culture models, Li et al developed a dynamic indirect 2D co-culture model using a transwell insert as a spacer [89]. MC3T3-E1 pre-osteoblasts in the lower compartment were subjected to mechanical strain by using a custom-built four-point bending device. The implementation of mechanical load promoted osteoblast differentiation (increase in ALP activity and expression) while their secreted soluble factors decreased osteoclasts differentiation of RAW264.7 cells in the top compartment (decrease in resorptive activity). Thus, the authors were able to reproduce the known osteogenic effect of mechanical load found in vivo [98] in an in vitro co-culture system. The addition of an osteocyte-like cell type into this co-culture model would be an interesting aspect as osteocytes are suggested to be the major load sensing cell type that orchestrates mechanotransduction [99,100].

In 2016, Pagani et al developed a tri-culture model of human endothelial cells (HUVEC), osteoblast-like cells (MG63) and osteoclast precursor cells (Poietics™ osteoclast precursors) [88]. Endothelial cells were placed in the peripheral area of a 12-well plate while the osteoclasts precursors were seeded on a 24-well glass slide that was then placed in the middle of the 12-well plate. Osteoblasts were seeded on a transwell cell culture insert and indirectly cultured on top of both aforementioned cell types. The results of the study showed that endothelial cells exerted a regulatory role on the osteoblast and osteoclast activity and differentiation in the tri-culture compared to mono- or bi-cultures after two weeks in vitro. This type of culture model allows the study of direct interactions between osteoclasts and endothelial cells and the indirect effects of and on osteoblasts. Importantly, the cell deposition on different substrates allows the separate analysis of each cell type even if they are directly cultured together.

#### 2.2.2. 2D Direct Co-Culture Models

In contrast to indirect models, direct cell culture models allow for direct cell interactions and, therefore, the study of juxtracrine signalling and its implications during bone remodelling. They are usually set up by simply seeding bone-building and bone-resorbing cells in the same culture vessel [90,91,101,102,103,104,105,106].

Takahashi et al developed a direct co-culture model using primary mouse calvariae osteoblasts and mouse spleen cells to investigate the effect of osteoblast contact on osteoclast formation [91]. The results showed that bone-resorbing osteoclast-like cells only developed when both cell types were cultured in contact during treatment with 1α,25-dihydroxyvitamin D3, which stimulates the release of RANKL by osteoblasts. When the cell types were separated by a semi-permeable membrane, osteoclast formation could not be observed, indicating that direct contact with osteoblasts is necessary for osteoclast maturation under these cell culture conditions.

Zhao et al used a direct co-culture of murine osteocyte-like MLO-Y4 cells with bone marrow cells to examine if direct contact of osteocytes can enhance osteoclast formation and activation [104]. Direct co-culture supported the formation of TRAP multinucleated cells from bone marrow cells, while the co-culture with less mature osteoblastic cell lines (2T3, OCT-1, or MC3T3-E1) also did not lead to the differentiation of osteoclasts. Importantly, treatment with conditioned medium did not lead to osteoclastogenesis. This study underlines the juxtracrine modulatory role of osteocytes during bone remodelling as activators of osteoclasts and thus bone resorption.

Another established co-culture model consisting of RAW 264.7 cells seeded on top of a ST-2 monolayer was used by Chen et al to investigate the effect (-)-epigallocatechin-3-gallate on osteoclastogenesis [107]. As the direct co-culture with bone marrow stromal cells such as ST-2 supports osteoclast differentiation, this model is suited to evaluate potential modulators of this differentiation process. The results of this study indicated that (−)-epigallocatechin gallate attenuates osteoclast differentiation (number of TRAP (+) stain osteoclasts and TRAP activity).

Schmid et al cultured hMSC-derived osteoblasts or undifferentiated MSCs together with human monocytes on bone slices to explore the formation of osteoclasts [106]. Without the addition of osteoclastic growth factors, osteoclastic cells formed in the co-culture with osteoblasts (TRAP and cathepsin K expression and actin ring morphology). However, the stimulation with M-CSF and RANKL was needed to render the osteoclast-like cell functional (resorptive activity). The study showed that the generation of osteoclasts, as indicated by common markers such as TRAP, does not necessarily lead to the development of osteoclastic cells with physiological function. Thus, the study also underlined the importance of using resorbable substrates in order to develop and detect functional osteoclasts for in vitro models of bone remodelling.

Hayden et al developed silk hydroxyapatite films as bone-like substrates for the co-culture of human MSC-derived osteoblasts and THP-1-derived osteoclasts [108]. Using quantitative surface metrology characterisations methods, they showed that the cells were able to remodel the substrate and that direct co-culture increased remodelling activity in comparison to mono osteoblasts cultures. In a follow-up study, the authors showed the suitability of this model for drug testing purposes, as they could measure the anti-resorptive effect of bisphosphonate loaded composite films on osteoblast–osteoclasts co-cultures [109].

Domaschke et al generated a 2D remodelling model by seeding osteoblast- and osteoclast-like cells on mineralised type I collagen membranes consisting of 30% collagen (*w*/*w*) and 70% (*w*/*w*) hydroxyapatite [110]. Similar to the study of Bernhardt et al, the substrate aimed to closely mimic the native bone extracellular matrix (ECM) [111]. Osteoblasts and osteoclasts differentiated from the precursor cells under osteogenic and osteoclastic stimulation and were able to remodel the bone-like membranes, which indicates the potential of this method to study bone remodelling in vitro.

An SaOS-2 osteoblast-derived ECM was used by Schulze et al to mimic the bone environment in their direct and indirect co-culture model [90]. In the direct co-culture, human osteoclast and osteoblast precursors were sequentially seeded on the matrix, while for the indirect model, the osteoblast precursors were placed in a transwell insert or conditioned medium was exchanged between the respective monocultures. Their results showed that only during direct co-culture, osteoclast-like cells developed without the addition of any osteoblastic or osteoclastic growth factors. The model was validated by the application of established anti-resorptive drugs (alendronate and zolendronate), which inhibited the resorptive activity of osteoclasts.

Young et al co-cultured human BMSCs and PBMCs on nanotopographical patterned polycarbonate substrates [112]. Their results showed that the nanotopographical modification led to spontaneous osteoblast and osteoclast differentiation of the precursors (as demonstrated by qPCR and histochemical analysis) without any further growth factor supplementation. This study showed that surface properties, such as surface roughness, provide important cues for the maturation of bone precursor cells and thus are critical for an authentic recapitulation of the remodelling process in vitro. However, the authors did not investigate the resorptive activity and thus functionality of the obtained osteoclasts.

Kim et al developed a direct dynamic co-culture model that aimed to mimic interstitial fluid flow found in bone in vivo. ST-2 cells and RAW 264.7 were cultured on a glass slide and placed in a flow chamber to subject the cell to oscillatory fluid flow [105]. Their results showed that less osteoclast-like cells were formed in the dynamic culture system compared to statically cultured cells, which stressed the major role of mechanical load in bone remodelling.

2D co-culture models in general greatly benefit from the variety of available methods and assays that can be used to analyse the cellular responses during stimulation with compounds of interest. In terms of bone remodelling research, the use of bone-like substrates [84,108] provides the cells with extracellular cues while allowing the visualisation of the activity of cells by microscopy. This is an advantage over 3D models in which the visual readout is obscured by the matrix or scaffold. 2D indirect models allow us to analyse both cell types separately due to their spatial division and thus permit the investigation of paracrine effects and analysis of cell type-specific responses to compounds of interest. Even though it has been suggested that osteoblasts and osteoclasts are locally separated during the remodelling process [5], recent research has shown that the resorptive activity of osteoclasts is regulated by osteoblasts through direct contact interaction in living bone [113]. Thus, 2D direct models may offer more physiological relevant results than indirect models due to the direct osteoclast and osteoblast interaction as in living bone. On the other hand, direct co-culture prevents the separate analysis of the respective cell types.

Cells that are cultured on 2D flat substrates, including biomimetic composites, are not provided with the environmental cues found in complex 3D extracellular environments such as substrate curvature [114]. Also, 2D cell culture leads to morphological changes as well as changes in the expression of proteins [115]. Cell polarisation, meaning the asymmetric spatial distribution of molecules within a cell to form functional domains [116], is disturbed in 2D culture [115]. The cell top surface, which is exposed to the culture medium, can form clusters of cell surface receptors through the abundant growth factor supply, while the bottom side facing the culture dish may have less stimulation to do so [115]. These intra- and extracellular cellular gradients lead to strong polarisation and results in aberrant autocrine and paracrine signalling activities affecting the cell phenotype [115]. The effect of 2D culture on cell phenotype and function have been showcased by a study by Gomez-Lechon et al, who showed that hepatocytes lose the expression of drug-metabolising enzymes in only a few days of culture in flat plastic dishes, while their enzymatic cellular function could be maintained for several weeks when they were kept in a collagen hydrogel [117].

#### 2.3. 3D Co-Culture Models

**Figure 3:**
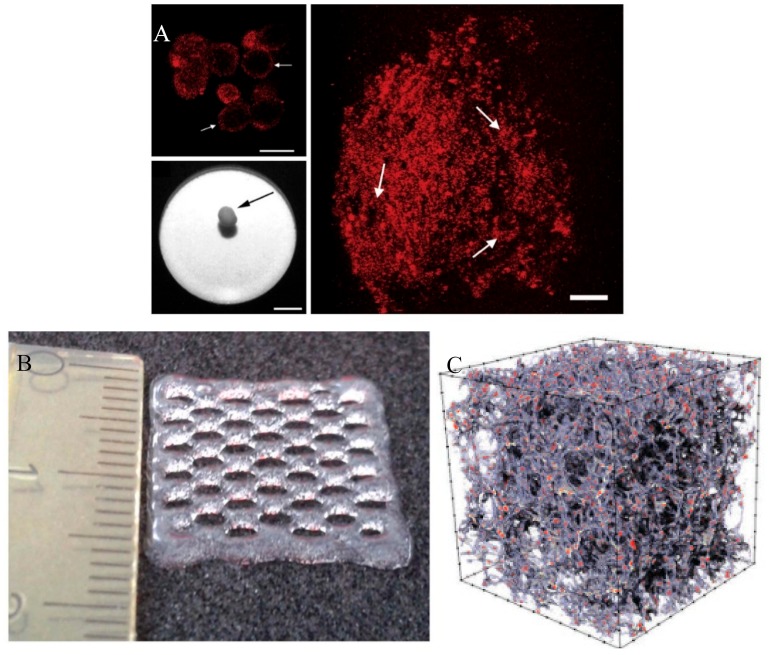
Illustration of exemplary (**A**) scaffold-free, scale bar 250 µm [118], (**B**) matrix-based (approximately 1.5 cm square) [119] and (**C**) scaffold-based (CT image with 3 mm edge length) [120] 3D co-culture models.

3D co-culture models aim to replicate the complex cell arrangement found in vivo by culturing cells on three-dimensional substrates. Commonly used types of substrates are soft matrices such as gels and cellular solids called scaffolds, while dynamic bioreactor-based culture systems can be used to establish scaffold/matrix-free 3D co-cultures (Figure 3).

Regardless of the chosen substrate type, it should imitate the native bone environment as close as possible to offer the cells a suitable microenvironment. Therefore, commonly chosen substrate materials incorporate hydroxyapatite and type I collagen as they are the major constituents of natural bone.

#### 2.3.1. Scaffold-Free

Mandatori et al used a rotary cell culture system to establish a scaffold-free co-culture of osteoblast precursors (human amniotic fluid MSCs) and osteoclast precursors (human monocytes) [121]. The cells formed spheroids containing both cell types and differentiated into osteoblast- and osteoclast-like cells over time, which allowed the study of the effects of candidate compounds on the formation of osteoclasts and osteoblasts.

Similarly, Clarke et al used a scaffold-free rotational co-culture system using primary adult human osteoblast and osteoclast precursors [118]. The histological analysis of those spheroid tissue constructs (>4 mm diameter) after 21 days showed the formation of osteocytes embedded in a mineralised matrix in the core region, which was surrounded by osteoblasts and osteoclasts. mRNA expression analysis showed that the relative production of certain osteogenic proteins (BMP-7 and BMP-2) was equal to adult human bone specimens.

The use of osteoblast and osteoclast spheroids as a co-culture model is advantageous due to its simple set up and it consequently has the potential to be used in high throughput applications. Also, this type of co-culture model can be easily expanded through the further inclusion of other cell types such as endothelial cells or immune cells if cell aggregation between the cell types is possible. Due to the lack of a 3D substrate, visualisation and optical analysis with fluorescence-based assays are possible, which is an advantage over scaffold-based models. As a consequence, more image-based analysis methods can be applied in comparison to other types of 3D models.

One of the main general limitations of scaffold-free in vitro models is the neglect of cell–ECM interactions during the model setup. It has been shown that cell–ECM interactions are essential for the cell polarisation and differentiation process [122,123,124]. For example, Han et al found that the cell fate of human MSCs into the osteogenic lineage can be guided through the spacing of cell-adhesive RGD peptide sequences on the culture substrate [124]. Smaller spacing enhanced the number of focal adhesions at the cell–substrate interface, increasing tension forces on the cytoskeleton, which ultimately lead to osteogenic differentiation. Also, substrate stiffness plays an important role in guiding the differentiation of precursor cells to a mature cell type [122,124] and in terms of bone models, stiff substrates are advantageous as they mechanically mimic the bone environment. Another disadvantage of scaffold-free cultures is the restricted nutrient supply and waste removal to the cells, which are localised in the core of the aggregates. Also, the variability in terms of internal organisation, shape and spheroid size have negative effects on model reproducibility [125].

#### 2.3.2. Matrices

Heinemann et al generated a 3D co-culture model of osteoblasts and osteoclasts on xerogel discs consisting of silica, collagen, and calcium phosphate [126]. Human bone marrow cells were seeded on the porous discs and kept in osteogenic medium before monocytes were added to the culture. Their results showed that osteoclast differentiation of the monocytes could be initiated without the addition of any osteoclastic growth factors and underlined the importance of direct crosstalk between the two cell types.

Panzavolta et al developed an indirect 3D co-culture model based on a strontium–hydroxyapatite–gelatin composite gel as a matrix [120]. The porous gel, fabricated by a freeze-drying process, was seeded with MG63 osteoblast-like cells and then placed in a well containing human osteoclast precursor cells (2T-110). Their results showed that the continuous release of strontium from the gel promoted osteoblast differentiation (as from gene expression analysis of marker genes and immunoenzymatic analysis of cell culture supernatant) and weakened osteoclast differentiation (TRAP staining and cell proliferation analysis).

Similar to the study of Schulze et al in 2D [90], Krishnan et al generated a naturally-derived collagenous matrix in 3D by culturing pre-osteoblastic cells for 60 days in a dynamic bioreactor culture system [127]. The secreted matrix was then seeded with pre-osteoblastic and osteoclastic cells to set up the remodelling model. Immunohistochemical analysis showed the formation of functional osteoclasts as indicated by digested collagen matrix, while osteoblasts were embedded in the matrix.

Bongio et al developed a tetra-culture model by encapsulating human umbilical endothelial cells, bone marrow MSCs, osteoblast and osteoclast precursors in a hydrogel containing collagen, fibrin and calcium phosphate nanoparticles [128]. The results indicated that both osteoblast and osteoclast differentiation were promoted (increased ALP activity and mineral deposition and increased TRAP activity and phosphate release respectively) in the tetra-culture enriched with calcium phosphate nanoparticles, in comparison to osteoblast/clast co-cultures without nanoparticles.

In contrast to the above-mentioned studies in which the in vitro models were manually fabricated, Zehnder et al developed a co-culture model using an automated fabrication approach [119]. Three-dimensional grid structures were fabricated by using an extrusion bioprinter depositing an alginate dialdehyde-gelatin gel containing ST2 and RAW 264.7 cells. The co-culture showed decreased ALP activity in comparison to ST2 monocultures but expressed more osteopontin, a late differentiation marker of osteoblasts. Osteoclast differentiation was also promoted, as evidenced by increased TRAP protein expression.

The use of matrices as substrates allows for a comparatively wide number of available imaging analysis methods due to the low visual interference of gels. Also, the low mechanical strength of gels allows easy access and thus the analysis of the inner parts. Growth factors or ECM components such as collagen can be readily incorporated into the matrices during the fabrication process to guide cell differentiation and maturation. The incorporation of candidate compounds raises the potential for drug screening applications. Importantly, the use of automated deposition techniques such as extrusion-based additive manufacturing (AM) techniques increase the scalability and reproducibility of matrix-based in vitro models. However, fabricated constructs lack vascularisation, and so the supply of nutrients and oxygen relies on passive diffusion through the gel, which is typically limited to distances of 100–200 µm. This limits the size of constructs that can be generated and perform effectively [129,130]. As a consequence, the development of novel techniques to generate vascularised 3D culture models is a major focus of tissue engineering scientists. Recently developed methods such as sacrificial ink writing are offering a solution to this problem [131]. However, one of the main limitations for the development of bone-related 3D models is that the soft extracellular surrounding does not match the elastic properties of the natural bone environment. It has been shown that the elastic modulus of the culture substrate provides essential cues to guide osteogenic differentiation [122]. Another major limitation of soft matrices is the lack of macro porosity, meaning pore sizes within a 100–300 µm range, which also stimulate bone cell maturation and proliferation [132].

#### 2.3.3. Scaffolds

Tortelli et al developed a 3D in vitro bone model by seeding murine osteoblasts and osteoclast precursors on a commercially available porous ceramic scaffold (Skelite) and compared cell maturation and mineral deposition to standard 2D co-cultures [133]. They could observe enhanced osteoblast differentiation (Runx2, osterix and osteocalcin mRNA expression), which was followed by promoted osteoclast differentiation (TRAP and cathepsin K mRNA expression). Histologic analysis of the sectioned scaffolds indicated promoted bone matrix deposition in the 3D environment, which together with the biochemical cellular analysis, showed that the 3D co-culture in this model enhanced cellular maturation in comparison to 2D cultures.

Jeon et al manufactured porous composite scaffolds consisting of poly-L-lactic acid, poly-lactide-co-glycolide and hydroxyapatite by salt-leaching [134]. Human-induced pluripotent stem cells derived -MSCs and –macrophages, respectively, preconditioned to the osteoblastic and osteoclastic lineages, were co-cultured on these scaffolds. Their results showed that the co-culture on scaffolds containing a comparably higher hydroxyapatite amount (5%) led to enhanced matrix deposition and the differentiation of more mature osteoblasts and osteoclasts after three weeks.

In 2011, Papadimitropoulos et al generated a dynamic tri-culture system of human osteoblast-, osteoclast- and endothelial precursor cells within a 3D porous ceramic scaffold [135]. The authors used a perfusion bioreactor system for cell seeding and cultivation of the tissue construct. In addition to histological staining methods, the analysis of the remodelling activity was carried out using non-invasive supernatant analysis (measurements of C-terminus procollagen type I, crosslinked N-telopeptides of collagen type I, phosphate concentration and TRAP isoform 5b content). The use of these non-destructive techniques allowed the remodelling activity to be efficiently assessed over a time course of interest without sacrificing samples at each time point. The results of the study indicated enhanced cell maturation as well as remodelling activity in the tri-culture system compared to the bi- or monocultures.

Nakagawa et al created a 3D bone remodelling model on polymer scaffolds in a dynamic bioreactor system [136]. The hydrophobic PLGA scaffold was coated with type I collagen to allow cell attachment and simulate bone ECM. Differentiated osteoblasts and osteoclast precursor cells were sequentially seeded on the scaffolds and maintained for 14 days. Histological and microscopic examination showed that the scaffold was mineralised by osteoblasts and that osteoclastogenesis occurred throughout the scaffold without the addition of osteoclastic growth factors.

Morelli et al fabricated porous composite polylactic acid-hydroxyapatite scaffolds by electrospinning [137]. Similar to the results of Bongio et al, they showed that the co-culture of osteoblasts and osteoclasts, as well as the addition of hydroxyapatite to the scaffold, promoted cell differentiation (as determined by ALP activity, cathepsin K expression and TRAP activity).

Midha et al co-cultured osteoclast and osteoblast precursors on bioactive glass foam scaffolds [138]. The authors showed that osteoclast-like cells were formed, with the ability to resorb the scaffold under osteoclastic growth factor stimulation while osteoblasts were depositing mineral matrix. Furthermore, their results indicated that the degradation products of the scaffold promoted cell maturation and proliferation.

Gamblin et al generated a 3D co-culture model by seeding human bone marrow derived MSCs on biphasic calcium phosphate particles [139]. The MSCs differentiated to osteoblast-like cells under osteogenic growth factor stimulation and connected the particles to a 3D structure through the deposition of collagenous ECM. These small bone-like tissue constructs were formed in 96-well plates within 83 days of culture. Then pre-osteoclast-like cells were seeded on these constructs and cultured for seven days under simultaneous osteogenic and osteoclastic stimulation. The presented results showed the formation of multinucleated osteoclast-like cells (evidenced by osteoclastic marker gene expression such as TRAP and cathepsin K) and osteoblast-like cells (increased ALP, type I collagen and bone sialoprotein gene expression) on the mineralised scaffolds.

Using solid scaffolds allows the use of calcium phosphate ceramics, which have been shown to support bone regeneration. For example, the use of bioactive glass scaffolds (composed of silica, sodium, calcium, and phosphate) supports bone cell differentiation and proliferation in vitro as well as osseointegration in vivo [140,141]. Next to supporting cell differentiation and proliferation, the right scaffold can also support cell colonisation and migration, consequently acting as a major driving force during bone in vitro tissue formation. Also, surface functionalisation through grafting or incorporation of growth factors or other molecules to the built material is possible, which can be used to further promote osteogenesis or to test the effect of candidate compounds [142]. Furthermore, the scaffold can be designed to incorporate a specific range of pores sizes to stimulate the desired cell response. In terms of bone cells, it has been shown that pore sizes around 300 µm lead to promoted osteogenesis in vitro [132].

Homogenous cell distribution throughout the scaffold is a critical aspect as cell density is an important factor during cellular differentiation and proliferation. The initial cell seeding process of the scaffold can be problematic due to the restricted access to the inner part of the scaffold. Different seeding strategies to avoid this problem are repeated injection seeding cycles, magnetic seeding or placement of seeded constructs on shakers [143,144]. However, these rely on manual operation and are therefore subject to low reproducibility and throughput. Models in which scaffolds are produced by osteoblasts themselves suffer from decreased reproducibility and the rather long matrix deposition process, which, for example, in the study of Gamblin et al, takes 83 days [139]. Moreover, fabrication of the scaffold by common methods such as freeze drying, gas foaming, electrospinning or salt leaching, do not allow independent control of morphological parameters (porosity, shape, interconnectivity). The random based deposition of material leads to intra- and inter batch variations concerning scaffold morphology and thus again to reduced reproducibility. The emergence of AM methods, including fused deposition modelling or stereolithography, offers the potential to overcome these issues as the built material is selectively deposited. However, additive techniques suffer from limited material choice. To opt for solid 3D scaffolds as a substrate also impairs the use of optical analysis methods due to the limited light path penetration. A further major limitation of scaffold-based osteoblast–osteoclast co-culture models is the dependency on destructive analysis by histological staining techniques to assess the remodelling activity.

## 3. Discussion

The use of osteoblast and osteoclast co-culture models allows us to study the interactions of both cell types in a simplified in vitro environment. The model types presented above are associated with different strengths and weaknesses and as a consequence have varying potential to meet key criteria for the successful development of new preclinical drug testing models (Table 3)

The improved conservation of cell phenotypes and functions, as well the presence of drug penetration barriers, make direct 3D in vitro co-culture models a suitable option to screen cell responses of human osteoblasts–osteoclasts to candidate compounds. Due to their shortcomings in terms of reproducibility, throughput and availability, human bone explants do not represent a viable option for drug testing purposes in an industrial setting. Nevertheless, they offer the highest physiological relevance of all in vitro model types and are thus suited to the study of basic bone biology. 2D models, whether direct or indirect, do not offer physiologically relevant cellular systems that are suited for preclinical drug screening even though their simple set up potentially allows for high throughput applications.

Although the shortcomings of 2D models and their inferiority to 3D tissue models are widely known, the translation of 3D models into the drug discovery process has been limited. 2D monocultures of bone cells in combination with subsequent animal testing remain dominant during the preclinical testing of identified osteoporosis drug candidates [23,144,145]. This is mainly due to the simple setup and high throughput of 2D cultures, which allows many compounds to be tested in a short period of time before candidates are tested in vivo [23].

Several challenges must be overcome to achieve the translation of 3D osteoblast/osteoclast co-cultures from basic research in academia to an industrial preclinical drug screening setting. For drug screening applications, osteoblasts and osteoclasts must be functional and not only phenotypically characterized by the expression of marker proteins. This is especially true for osteoclasts, which are often only described by the expression of TRAP and the presence of multiple nuclei. It has been shown that this does not necessarily render the cells functional, which is of major importance when the remodelling activity is assessed. Many of the co-culture studies utilise primary human PBMCs or murine RAW 264.7 as osteoclast sources in their model set up. However, during the initial model development phase, where reproducibility is fundamental, the use of human cell lines instead of PBMCs is advantageous due to the elimination of cell donor variability, which increases reproducibility. The use of the prominent murine RAW 264.7 cell line may increase reproducibility but dampens the translational aspect of the obtained results. There are several human osteoblast-like cell lines (e.g., SaOs-2, hFOB 1.19) readily available but so far none have been established for osteoclast-like cells. Here, human monocytic cell lines such as THP-1 or U937 can serve as a consistent osteoclast precursor cell source [146,147] and therefore should be considered during the first steps of model development. As the translational aspect of cell lines is very limited, they should be replaced by human primary cells once the developed model has been validated so that drug candidates can be tested on clinically relevant cells and across a representative number of donors.

Once the functionality of both cell types is ensured, the fabrication method of the substrate should be automated and standardised. So far, many of the 3D models are fabricated by time-consuming fabrication methods (e.g., freeze drying, gas foaming, electrospinning or salt leaching), which rely on the random deposition of substrate material and require substantial manual operation. This reduces throughput and leaves room for human error. Together, this leads to inter- and intra-batch variations and reduces reproducibility. Here, the emergence of automated manufacturing methods such as AM or melt electrowriting, which allow selective deposition of substrate materials, offer a solution for the standardised fabrication of 3D substrates. Automated manufacturing methods will also cut the comparably long production time of a model single specimen, improving the scalability and throughput.

Also, the final characterisation and analysis step of 3D models must be automated to increase the throughput further. In vitro, the net remodelling activity of osteoblasts and osteoclasts is the most relevant metabolic parameter for assessing the effect of candidate compounds (comparable to bone mineral density measurements in vivo) as it decides over the balance of bone synthesis or resorption. Once a candidate has been identified by this major metabolic parameter, research on the mechanism (e.g., involved signalling pathways) by which the remodelling activity is affected may also be conducted, depending on the aim of the study.

Up until now, there is no fast and reliable method to determine the net remodelling effect in 3D in vitro and, therefore, the development of new and automated monitoring methods to analyse the balance between bone formation and resorption must be addressed for the compound screening process. The problem of assessing bone matrix turnover in 3D models was, for example, addressed by Ruggiu et al who used X-ray computed microtomography to quantify the bone matrix turnover of osteoblasts and osteoclasts cultured on 3D scaffolds [148]. But even though this analysis method is comparably cheap, it is not suited for high throughput applications due to the long duration that high resolution scans require. Potential solutions to achieve high throughput during the analysis is the integration of electrochemical and/or optical microsensors systems within the 3D model. For example, sensors have been already developed and established to analyse cell metabolism through the measurement of released substances in the culture medium [149].

In summary, to successfully use 3D osteoblast/osteoclast co-culture models for preclinical drug testing purposes, the use of human-derived cells, with direct monitoring of the net remodelling activity, is needed. This can be achieved through the integration of automated fabrication methods such as AM in combination with automated analysis methods such as microsensors or automated imaging modules.

## 4. Conclusions

3D models suffer in general from low throughput, a rather complicated setup and low reproducibility. Nevertheless, 3D co-culture models of osteoblasts and osteoclasts have the potential to enhance the drug discovery process for bone remodelling associated diseases during the preclinical stage. Currently, fabrication and characterisation of 3D models are performed manually and thus are time-consuming and do not allow for high throughput. Further automatisation of the fabrication and analysis process may promote the reproducibility and throughput of these models, making them a valid alternative to animal models for preclinical drug screening purposes.

## Figures and Tables

**Table 1:** Overview of the major available osteoporosis animal models.

Osteoporosis Type	Postmenopausal Osteoporosis	Disuse Osteoporosis	Glucocorticoid-Induced Osteoporosis
**Induction Method**	Ovariectomy	Hind limb immobilisation Tail suspension	Glucocorticoid treatment
**Animal**	mouse rat sheep non-human primates	mouse rat dog	mouse rat rabbit dog sheep

**Table 2:** Summary of literature on bone tissue cultures, divided by field of application (**A**) or by origin of bone (**B**).

**A.** ** Field of Application**	
Cancer research	Breast [26,27,28,29,30,31,32,33,34]
	Prostate [28,30,33,34,35,36]
	Multiple myeloma [37]
	Fibrosarcoma/osteosarcoma [38]
Method development	Culture type:Perfusion [39,40]Static [41,42,43,44,45]Static—embedded in agarose [46,47]Combined perfusion and loading [48,49]
	Assessment of viability [50,51]
	Assessment of bone formation [52]
Bone biology research	Bone fracture repair/endochondral ossification [46,53,54,55,56]
	Bone formation and/or remodeling [47,57,58,59,60,61,62,63,64,65,66,67,68]
	Bone response to load/strain/microgravity/other biophysical stimuli [47,59,60,69,70,71,72,73,74,75,76,77]
	Bone development and basic biology [78,79,80,81,82,83]
**B.** ** Derivation of Bone**	
Human	[31,33,40,48,49,50,71]
Murine	[26,27,28,29,30,32,34,35,36,37,38,41,43,44,45,52,53,56,57,58,61,62,63,64,65,67,75,76,77,78,79,80,81]
Bovine	[40,43,48,49,50,59,72,73,76]
Rat	[39,46,47,60,68,69,74]
Chicken	[42,54,55,77,82,83]
Ovine	[48,49,50]
Porcine	[51]
Atlantic cod	[66]

**Table 3:** Key criteria for preclinical drug testing models. Legend: +++ meets key criterion; ++ partially meets key criterion; + meets key criterion to some degree; - minor limitations regarding criterion; - - more significant limitations regarding criterion; - - - does not meet key criterion.

Key Criteria	Reproducibility	Throughput	Physiological Cell–Cell and Cell–Matrix Interaction	Can Be Assessed Using Commonly Available Analysis Methods	References
2D indirect plastic substrate	+++	++	---	+++	[68,69,70,71,72,75,76,77,78,79]
2D indirect with bone like substrate	++	+	- -	+++	[67,73]
2D direct plastic substrate	+++	+++	- -	+++	[74,84,85,86,87,90]
2D direct bone like substrate	++	+	-	++	[73,78,88,89,90,91,92,93]
3D scaffold free	+	+	+	++	[101,104]
3D matrix	-	- -	++	- -	[102,103,110,111,112]
3D porous scaffold	- - -	- - -	+++	- - -	[117,118,119,120,121,122,123]

## Abbreviations

AM EU	Additive manufacturing European Union
US	United States
UK	United Kingdom
ALP	Alkaline phosphatase
hPBMC	Human peripheral blood mononuclear cell
MSC	Mesenchymal stem cell
RANKL	Receptor activator of nuclear factor-kb ligand
M-CSF	Macrophage colony-stimulating factor
TRAP	Tartrate-resistant acid phosphatase
hMSCs	Human mesenchymal stem cells
ECM	Extracellular matrix
RGD	Arginine, Glycine, Aspartate
PTH	Parathyroid hormone
DNA	Deoxyribonucleic acid
sFRP-1	Secreted frizzled-related protein 1
DMEM	Dulbecco's Modified Eagle Medium
qPCR	Real-time polymerase chain reaction
RUNX2	Runt-related transcription factor 2
ELISA	Enzyme-linked immunosorbent assay
LDH	Lactate dehydrogenase
BMP	Bone morphogenetic protein
